# Front and center: Maturational dysregulation of frontal lobe functional neuroanatomic connections in attention deficit hyperactivity disorder

**DOI:** 10.3389/fnana.2022.936025

**Published:** 2022-08-23

**Authors:** Gerry Leisman, Robert Melillo

**Affiliations:** ^1^Movement and Cognition Laboratory, Department of Physical Therapy, University of Haifa, Haifa, Israel; ^2^Department of Neurology, University of Medical Sciences of Havana, Havana, Cuba

**Keywords:** ADHD, frontal lobe, prefrontal cortex, indirect pathway, direct pathway, hyperdirect pathway

## Abstract

Frontal lobe function may not universally explain all forms of attention deficit hyperactivity disorder (ADHD) but the frontal lobe hypothesis described supports an internally consistent model for integrating the numerous behaviors associated with ADHD. The paper examines the developmental trajectories of frontal and prefrontal lobe development, framing ADHD as maturational dysregulation concluding that the cognitive, motor, and behavioral abilities of the presumptive majority of ADHD children may not primarily be disordered or dysfunctional but reflect maturational dysregulation that is inconsistent with the psychomotor and cognitive expectations for the child’s chronological and mental age. ADHD children demonstrate decreased activation of the right and middle prefrontal cortex. Prefrontal and frontal lobe regions have an exuberant network of shared pathways with the diencephalic region, also having a regulatory function in arousal as well as with the ascending reticular formation which has a capacity for response suppression to task-irrelevant stimuli. Prefrontal lesions oftentimes are associated with the regulatory breakdown of goal-directed activity and impulsivity. In conclusion, a presumptive majority of childhood ADHD may result from maturational dysregulation of the frontal lobes with effects on the direct, indirect and/or, hyperdirect pathways.

## Introduction

We think that attention deficit hyperactivity disorder (ADHD) results from differences, when compared with the normally developing child, in the trajectory of cortical maturation and well as from deviations in the trajectory of asymmetric brain development (Rubia, 2007; Janssen T. W. P. et al., 2017; Bouziane et al., 2018; Ha et al., 2020). These developmental differences in the development of hemispheric asymmetries significantly relate to the expression of the characteristics of ADHD and can explain many of the symptoms that are evidenced (Ha et al., 2020; Chen et al., 2021; Postema et al., 2021). The condition speaks to the relationship between the functions of the hemispheres. Overactivity of the left hemisphere can lead to hyperactivity of movement and hyperkinetic behavior (Wasserstein and Stefanatos, 2016; Helfer et al., 2020). The right hemisphere is mainly responsible for attention especially sustained attention which is the main attentional deficit in ADHD (Longo et al., 2015; Bartolomeo and Malkinson, 2019). Therefore, underdevelopment of the right hemisphere is related to the attentional deficit (Zou and Yang, 2021). This hyperreactivity of one cerebral hemisphere combined with underdevelopment of contralateral hemisphere speaks to the nature of many neurobehavioral disorders (Melillo and Leisman, 2009; Douglas et al., 2018).

The beginning of the brain’s developmental interregional communication differences in ADHD as compared with neurotypical children has been thought to commence *in utero* or early in post-partum development (Hanć et al., 2018; Vizzini et al., 2019; Xi and Wu, 2021). The right hemisphere develops first in the womb and for the first 3 years (Uda et al., 2015; Caccappolo and Honig, 2016). Early childhood functional brain asymmetry has been confirmed by cerebral blood flow changes measured at rest between 1 and 3 years of age, blood flow studies demonstrate the predominance of the right hemispheric, largely associated with the activity in the posterior associative area (Paniukov et al., 2020). Asymmetry modulates to the left after approximately 3 years of age (Tzourio-Mazoyer et al., 2017). After 3 years of age, the time course of changes appears to follow the emergence of functions localized initially on the right, but later on the left hemisphere (i.e., visuospatial and later language abilities) (Spagna et al., 2016; Olulade et al., 2020). These findings support the hypothesis that, in human infancy and early childhood, the right hemisphere develops its functions earlier than the left (Chiron et al., 1997; Melillo and Leisman, 2010, 2015). The left hemisphere takes the lead in development for the next 3 years (Chiron et al., 1997; Melillo and Leisman, 2010, 2015).

This one-side-at-a-time developmental activity of the hemispheres is thought to be an important factor that is highly associated with the development and lateralization of the brain in infancy and early childhood (Melillo and Leisman, 2010). This asymmetry and lateralization impart great advantage to the brain as it leads to regional specialization which increases the efficiency of the brain (Duboc et al., 2015). The brain does not like redundancy as it renders its ability to communicate between regions less optimized and slows down the brain’s responsivity to internal and external stimulation and adversity (Hiratani and Fukai, 2018).

In order to speed-up brain responsivity to external or internal voluntary action control, fronto-basal ganglia pathways must play a significant role in the control of voluntary action and in motor response inhibition. Response inhibition can be facilitated by a fast hyperdirect pathway that would connect the right inferior frontal gyrus and the pre-supplementary motor area with the subthalamic nucleus or, through the indirect pathway between the cortex and caudate. These considerations are explored further below.

## Top-down and bottom-up communication in ADHD

The brain develops from the bottom up starting in the lower brainstem and with the brainstem nuclei acting as precursors to higher levels of brain development and with the ultimate development of Brodmann areas that have both structural and functional differences (Zelazo, 2015; Onofrj et al., 2022). Once there is bottom-up completion of development there then can be completion of top-down development which allows the brain and neocortex to ultimately control all functions of the body (Emberson et al., 2015). As part of this top-down development, the brain and especially the prefrontal cortex develops feedback pathways with the basal ganglia and thalamus that ultimately control and regulate much of human behavior (Petrovic and Castellanos, 2016; Emberson, 2017; Choi et al., 2018). There are at least five loops with connections from the prefrontal cortex to the basal ganglia and entering the direct or indirect pathways. The direct pathway is facilitatory and the indirect pathway, inhibitory.

### Direct, indirect, and hyperdirect pathways in ADHD

The original model by Alexander et al. (1986) described five feedback loops that included the promotor area [Broca’s Area (BA) 6] to control motor function, the dorsolateral prefrontal cortex (BA 9, 46) for executive function (EF), the frontal eye field (BA 8) for control of volitional saccadic eye movement, the orbitofrontal cortex (OFC) (BA 11, 12) for control of social behavior and the anterior cingulate (AC) (BA 24, 25, 32, 33) for control of motivation. Middleton and Strick (2000), however, created a revised version of this that expanded the number of feedback loops to seven motor subcircuits, three oculomotor circuits, four dorsolateral prefrontal circuits (DLPFC), five OFC circuits, and two cingulate circuits.

**FIGURE 1:**
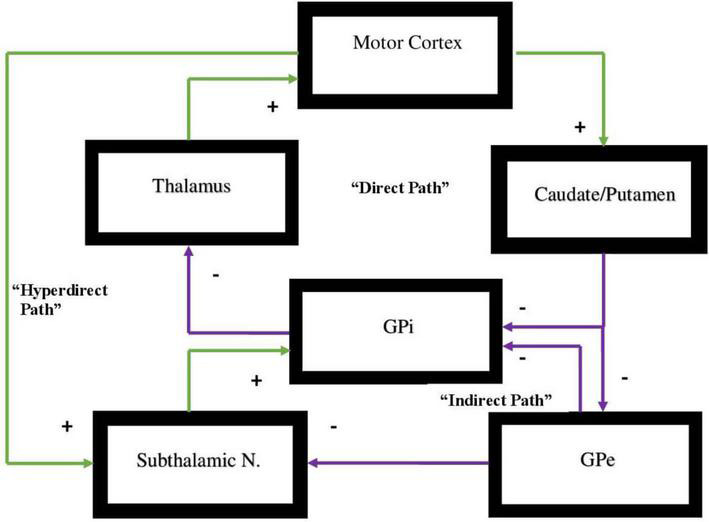
Representation of the direct vs. indirect pathways of the basal ganglia indicating facilitatory vs. inhibitory components of motor activity. In the direct pathway, Input from the cerebral cortex to the striatum is associated with triggering of inhibitory neurons in the striatum. This subsequently is associated with increased inhibitory output projecting to the globus pallidus-internal [GPi]. Subsequently, decreased inhibitory output from GPi to the ventral anterior [VA] and ventral lateral [VL] nuclei of the thalamus is evidenced that in turn projects through excitatory pathways to the premotor cortex. The direct pathway regulates motor and premotor cortical excitation that is involved in planning and movement initiation. The indirect pathway, when appropriately functioning, should inhibit movement when cortically generated excitatory activity enables inhibitory neurons in globus pallidus external [GPe]. These subsequently inhibit tonic inhibitory output neurons associated with decreased tonic inhibition of the subthalamic nucleus [STN]. The result is increased excitatory output to GPi. Excitatory input to GPi adds inhibitory output from GPi to the thalamus which, in turn, decreases excitatory feedback to cerebral cortex. The result, under normal circumstances, should lead to the inhibition of motor activity. Dopamine supports the activity of the direct pathway suppressing activity of indirect pathway. The hyperdirect pathway is exceptional as it circumvents the striatum with a direct link from the cortex to the subthalamic nucleus, then directing excitatory projections to the GPi. The hyperdirect pathway is key for containing non-purposeful movement. When the system is impaired, individuals are less able to inhibit unplanned motor activity.

All of these circuits project from a specific area of the cortex to the basal ganglia and from there to the thalamus then returning to the cortex (Zikopoulos and Barbas, 2007; Sherman, 2011). Each one of these circuits projects either to the indirect or direct pathways and will either activate or inhibit a specific behavior or function in the direct pathway or in the indirect pathway, respectively. Motor behavior is in large measure dependent on a dynamic balance between these two pathways where neither pathway gains dominance over the other (Cui et al., 2013; Macpherson et al., 2014; Hikosaka et al., 2019; Kwak and Jung, 2019). The pathways are represented in Figure 1.

There exists an additional pathway that plays a significant role in oscillating between direct and indirect pathways and is critical to this dynamic balance between these pathways and behavioral flexibility. This is termed the hyperdirect pathway and it originates from the right cerebral hemisphere alone (Koirala et al., 2018; Chen et al., 2020). There are two regions of the right hemisphere that are the points of origin of the hyperdirect pathway which specifically activates the indirect pathway at the caudate and putamen and specifically connects to the subthalamic nucleus of Luys, the main source of the indirect pathway’s effect (Chen et al., 2020; Temiz et al., 2020). The hyperdirect pathway has one component arising from the premotor area (BA 6) in the right hemisphere. This pathway primarily inhibits motor activity (Chen et al., 2020).

The hyperdirect pathway suppresses unwanted movement and it will subsequently inhibit movement once an action has been completed (Nambu et al., 2002; Chen et al., 2020). If there exists a motor activity deficit or underdevelopment of this pathway and its connections, overactivity of the premotor loop on the left hemisphere will likely be evidenced (Singer et al., 2015; Dalley and Robbins, 2017; Guo et al., 2018; Temiz et al., 2020; Sival et al., 2022), which will, in turn, activate the direct pathway and increase motor activity that can be exemplified by motor tics (Leisman and Sheldon, 2022), or stereotypical movements not infrequently evidenced in hyperkinetic disorders such as ADHD, Tourette’s syndrome, autism spectrum disorder (ASD), etc. (Melillo and Leisman, 2009; Temiz et al., 2020; Hannah and Aron, 2021). The other part of the hyperdirect pathway arises from the inferior frontal gyrus (BA 44, 45, 47) in the right hemisphere alone (Chen et al., 2020; Narayanan et al., 2020). This is thought to regulate the limbic, and associative loops, which includes the DLPFC, OFC, and the AC by specifically activating the indirect pathway to eliminate unwanted or inappropriate, emotions, social behavior, thoughts, etc. (Janssen M. L. et al., 2017; Temiz et al., 2020).

**FIGURE 2:**
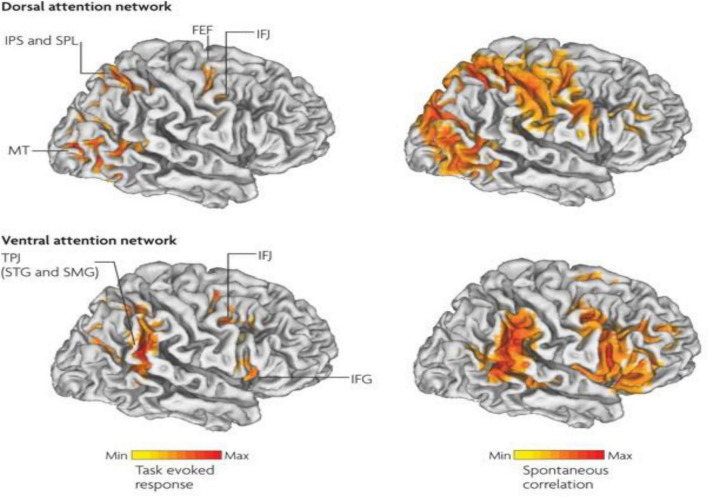
Interaction between the dorsal attention networks and ventral (salience) enables active control of attention in relation to bottom-up sensory stimulation and top-down goals. The top-down dorsal-frontoparietal system which includes the frontal eye fields (FEF) and the intraparietal sulcus that supports voluntary attention to particular aspect of the visual field locations or objects and the ventral-frontoparietal system is concerned with attention to unexpected features. The ventral attention network involves the ventral-frontal cortex and the temporoparietal junction (TPJ), and usually responds to behaviorally relevant but unexpected stimuli. The biasing of sensory areas toward particular stimuli derives from the frontoparietal cortex. There exists a connection between sensory cortical areas involving the intraparietal sulcus and the FEF. These two areas have top-down influences on the orienting of attention. These top-down effects are known to out-weigh bottom up effects from the visual cortex (after Vossel et al., 2014 with permission).

**FIGURE 3:**
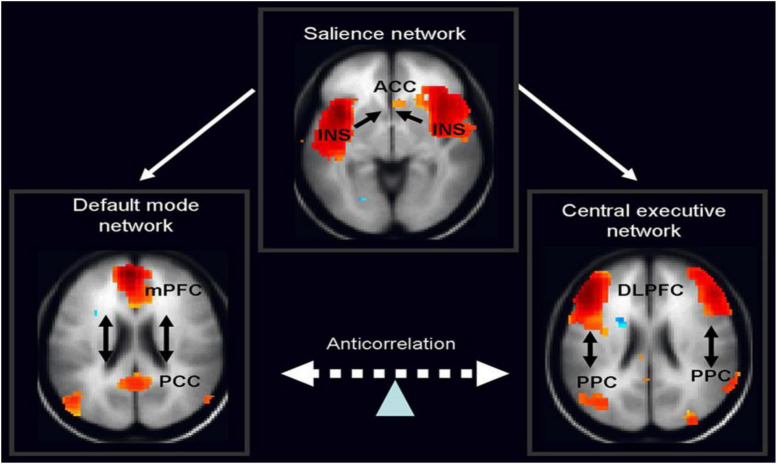
The salience network is theorized to mediate switching between the default mode network (DMN) and central executive network (CEN) (adapted from Vossel et al., 2014, with permission).

Therefore, in ADHD, we can see that many of the symptoms can be explained by overactivity of the left hemisphere’s connections to the direct pathway related to the underdevelopment and underactivity of the right hemisphere and the indirect and hyperdirect pathways (Chen et al., 2016; Hauser et al., 2016; Ziegler et al., 2016) This can explain the hyperactive motor behavior seen in ADHD with overactivity of BA 6 in the left hemisphere associated with underdevelopment of BA 6 on the right. This also can explain the underdevelopment of sustained attention abilities which is related to the ventral attention network, lateralized more to the right hemisphere and subserving sustained attention (Vossel et al., 2014) and is reflected in Figure 2. This is also connected to the salience network represented in Figure 3 which is predominately constituted by the insula cortex (IC) (BA 13) and the (AC) (BA 25,32) (Sridharan et al., 2008; Menon, 2011; Nekovarova et al., 2014). This developmental maturational imbalance between all of these loops can explain of the symptoms seen in ADHD.

## Central executive and default mode networks in ADHD: In support of goal-directed behavior

### Default mode network

Neuroimaging studies have led us to theorize that the fundamental differences between rest and agency can be based on an organized level of baseline activity that is diminished during goal-oriented cognition. It has also been thought that the brain maintains a “default mode” in the absence of cognitive demands (Gusnard and Raichle, 2001; Gusnard et al., 2001; Raichle and Gusnard, 2005) so as to enable a readiness state that is capable of responding to changes in one’s environment (Raichle et al., 2001). The Default Mode Network (DMN) is a network of coherent brain regions active during daydreaming or unfocused behavior. Some investigators have linked activity of the DMN to the processing of self-referential information as brain regions such as the posterior cingulate (PCC) and medial prefrontal cortex (mPFC) have been demonstrated to subserve self-reflection, introspective mental imagery, and self-awareness (Northoff et al., 2006; Buckner et al., 2008; Schneider et al., 2008).

A meta-analysis (Spreng et al., 2009) identified components of the DMN, such as the anterior cingulate cortex (ACC), the PCC, mPFC, and the middle temporal gyrus and. Central Executive Network (CEN) activation tasks have been reliably confirmed to stimulate decrease activation (deactivation) in the DMN. McKiernan et al. (2003) demonstrated that with increased task difficulty, task-related deactivation increased. Two studies by Fransson (Fransson, 2006; Fransson and Marrelec, 2008) examined DMN connectivity during challenging cognitive tasks and found significantly reduced functional connectivity within the DMN with excessive working memory load.

Different groups (Buckner et al., 2008; Spreng and Grady, 2010) have discussed the notion that the DMN might consist of numerous subsystems. Uddin et al. (2009, 2010) and Uddin (2021) showed significant differences by examining the anticorrelations of seed regions in the PCC and mPFC. This indicated that distinct nodes of the DMN may modulate activity in task-positive networks differently. Alterations in connectivity of the DMN have been discussed as possible biomarkers for psychiatric conditions such as autism (Calhoun et al., 2008). Specifically related to ADHD, Rubia et al. (2014), have noted that individuals with ADHD have greater gray matter volume in nodes within the DMN. When performing a task, the DMN activity infringes on the task-positive cognitive systems necessary for task completion (Rubia et al., 2014). We acknowledge that our personal DMN has been active when we suddenly return from having been “zoned-out” and realize it. When we engage in goal-oriented tasks that are attention-demanding, the DMN decreases its activity. Although in normal development, difficulties inhibiting or deactivating the DMN is likely, individuals with ADHD have significantly greater difficulty in inhibiting the DMN. In other words, individuals with ADHD have a stronger gravitational pull toward this cognitive resting state and, as a result, it requires significantly greater effort to gravitate away from it and attend to the task. Uddin et al. (2008) found reduced DMN nodal homogeneity in ADHD individuals when compared to age-matched controls, that was most evidenced between the precuneus and other DMN regions. This finding provides further support for the notion that altered precuneus connectivity is implicated in ADHD.

### Central executive network

The CEN is usually related to the appropriate functioning of the PFC and related regions such as the cingulate cortex (Cohen, 2017). The CEN has often been considered synonymous with the earlier concept of EF. In both, behavioral regulatory activity can optimize goal-directed behavior and prevent automaticity in a way similar to the difference between automatic and controlled responding (Schneider and Shiffrin, 1977). This approximately aligns with the distinction between habit and goal-directed responsivity (Balleine and O’Doherty, 2010). One would expect the absence of the CEN to produce automatic behavior as controlled responses are flexible and goal-directed.

Miller and Cohen (2001) thought that the CEN “…stems from the active maintenance of patterns of activity in the PFC that represent goals and the means to achieve them. They provide bias signals to other brain structures whose net effect is to guide the flow of activity along neural pathways that establish the proper mappings between inputs, internal states, and outputs needed to perform a given task” (p. 167). This conception of the role of PFC in the CEN basically consists of the contextual biasing of attention (e.g., instructions) to exert attentional control and to resolve conflicts. In a modified Stroop task, Kerns et al. (2005) found that the theory was supported by an fMRI study demonstrating that ACC activation was supplemented by activity in the DLPFC associated with top-down adjustments of response control. Therefore, in Miller and Cohen’s (2001) model, the ACC can identify conflict resolved by the top-down biasing of response options from the DLPFC. This theoretical scheme has provided support for a CEN process mediated by interactive PFC circuitry.

Both the CEN and DMN are lateralized (Sripada et al., 2014). The CEN tends to be more left (Silk et al., 2016) and more focused on the external environment (Antshel et al., 2014) which is overactive in ADHD (Bilevicius et al., 2018). The DMN tends to be more lateralized to the right (Sripada et al., 2014) and appears to be more internally focused (Lanier et al., 2021) the results of which are significant features of ADHD (Seli et al., 2015). Individuals with ADHD manifest a reduced connection to their bodies (Wiersema and Godefroid, 2018) as well as reduced sensory awareness of body parts (Sanz-Cervera et al., 2017).

Additionally, not only is there a reported decrease in pain perception (Wolff et al., 2016) as well as sensory perception to tactile (Puts et al., 2017) and proprioceptive stimulation (Tseng et al., 2018; Tarbanie, 2020), but individuals with ADHD also have reduced interoception (Kutscheidt et al., 2019) which is related to the functioning of the right insula and the salience network (Uddin, 2015; Zhang et al., 2019) which, in turn, is associated with the ventral attention network and sustained attentional function (Janssen et al., 2018). Salience also tends to be more lateralized to the right hemisphere (Uddin, 2015; Zhang et al., 2019). In addition, the left DLPFC supports setting goals (Vetter et al., 2018) and the left hemisphere is more active when sustaining goals OFC and goal intensity (Chiang et al., 2015), in turn, largely associated with the left hemisphere’s BA 44 (Pagliaccio et al., 2017).

### Developmental delay in neuroanatomic maturational dysfunction of the frontal lobes in ADHD

The frontal lobes exemplify a complex neurological system. The prefrontal cortex is integrated within the frontal lobes and is thought to combine intentional responses that require intended and synchronized action sequences (Laubach et al., 2015). Frontal lobe complexity is demonstrated by prefrontal cortex interconnectedness with the motor regions of the frontal lobes (Bernard et al., 2016), the posterior associative cortex (Barbas, 2015; Fuster, 2015), the limbic (motivational) (Barbas, 2015; Tucker and Luu, 2021), and ascending reticular activating system (arousal) (Jang and Kwon, 2015). These interconnections, in particular, with the dorso thalamic nucleus projections, describe the primary features of prefrontal cortical organization (Leisman and Melillo, 2012; Bubb et al., 2017; Kamali et al., 2020).

There are three classes of neuropsychological functioning associated with the prefrontal cortex: regulatory, social, and executive (Fuster, 2015). The prefrontal cortex supports the maintenance of set, in problem-solving tasks (Friedman and Robbins, 2022), and in implementing strategic and sequential planning (Desrochers et al., 2015; Schuck et al., 2015), performing mental representations of a task (Monk et al., 2021), planning and self-monitoring of performance (Joensson et al., 2015), abiding by social rules (Rozzi and Fogassi, 2017), and employing environmental cues (Fuster, 2015; Hall-McMaster et al., 2017). In adults with lesions of the frontal lobes, there exists evidence of impairment in action or response planning, anticipation of events, establishment of goals, self-monitoring ability, cognitive flexibility with comorbidities with conditions such as ticking behavior (Leisman and Sheldon, 2022) and other neurobehavioral disorders such as ASD and OCD (Melillo and Leisman, 2009). Frontal lobe lesioned adults present with disinhibition, perseverative behavior, and difficulty in employing environmental cues to modulate behavior (Fuster, 2015; Serrien and Sovijärvi-Spapé, 2015).

Frontal lobe lesions in adults allows us to observe hyperactivity control mechanisms more readily (Clay et al., 2019; Hagiescu, 2021). Hyperactivity, both in childhood and in adulthood, can be viewed as a disturbance of higher levels of cortical inhibition manifested as an absence of orienting responses inhibition (Posner et al., 1998; Brown et al., 2021; Williams and Das, 2021), an inhibitory deficit of inappropriate responses (Posner et al., 1998) and/or a disinhibition of inhibitory cortical reflexes (Neely et al., 2017), or retained primitive reflexes (Melillo and Leisman, 2010; Melillo et al., 2020; Bob et al., 2021; Sigafoos et al., 2021). Given the apparent similarity in the behavioral manifestations of ADHD and adults with dysfunction of or damage to the frontal lobe, we can hypothesize a common origin for ADHD and frontal lobe dysfunction, even though it has long been argued (Fletcher and Taylor, 1984, p. 46; cf. Fletcher, 2021), that, “Similarity of behavior in the absence of independent assessment does not provide sufficient evidence of common origins” in adults and children.

ADHD as a manifestation of maturational dysregulation has been largely supported by MRI studies. Volumetric measurements of right and left hemispheres, of gray and white matter within each lobe, and cerebral and cerebellar volume have been reported to be approximately 4% smaller in ADHD individuals relative to controls (Castellanos et al., 2002). Significant differences have also been noted in cortical thickness (Shaw et al., 2007). While in ADHD and control g children, peak cortical thickness was developed earlier in the sensory regions as compared to association cortical regions. However, control children developed peak thickness between 7 and 8 years, of age relative to ADHD children who attained it later, between 10 and 11 years. This evidence supports a common course of regional brain development sequencing in both ADHD and control children but with cortical maturational dysregulation in ADHD.

More evidence in support of widespread volumetric reductions in ADHD subjects comes from cross-sectional studies comparing ADHD and control subjects in smaller samples than in the above studies (see reviews Seidman et al., 2005; Shaw and Rabin, 2009). While there are many mixed findings in this body of work, the majority indicated that volumes were reduced in ADHD subjects relative to age-matched controls. The loci of the reported reductions are in multimodal association cortices such as the frontal lobes and its subregions, premotor cortex, posterior cingulate, anterior and medial temporal lobes, cerebellar lobules, and basal ganglia structures (caudate, globus pallidus, putamen, and ventral striatum).

Cognitive and motor affect assessment in the context of the frontal lobe hypothesis of ADHD has been partly obstructed by argument about the developmental stage at which functioning of the frontal lobes matures. Earlier, Luria (2012) had proposed that prefrontal regions are not capable of agency and preparedness for action until between of 4 to 7 years of age under normal circumstances. Golden, on the other hand (Bradley and Golden, 2001; Golden and Hines, 2010) noted that the frontal areas do not become functionally mature until much later, in adolescence. Since Luria and Golden, we have learned that frontal lobe behaviors develop rapidly from the age of approximately 6 years and almost reach adult levels of control between 10 and 12 years of age (Norbom et al., 2020; Wang et al., 2020).

## Conclusion

The issue of developmental trajectories is singularly important as it frames the disorder of ADHD as a maturational dysfunction. The result, therefore, is that the cognitive and behavioral abilities of the ADHD child are not disordered or dysfunctional, but are rather developmentally inappropriate for the child’s chronological and mental age.

Compared to neurotypical children, those with ADHD demonstrate decreased activation of the right and middle prefrontal cortex across all age groups (Yasumura et al., 2019). However, while frontal lobe function may not universally explain all forms of ADHD, the frontal lobe hypothesis described here does provide an internally consistent model for the elucidation of many of the findings associated with ADHD. Prefrontal regions of the frontal lobes have an exuberant network of shared pathways with the diencephalic region (Bubb et al., 2017), which has a regulatory function in arousal (Martella et al., 2020), as well as with the ascending reticular formation which, for reasons previously indicated, has a capacity for response suppression to task-irrelevant stimuli. Prefrontal lesions oftentimes are associated with regulatory breakdown of goal-directed activity and impulsivity. Individuals with frontal and prefrontal lesions have an impediment in subduing ongoing activities independent of environmental feedback and demonstrate amplified responsiveness to extraneous stimuli (impulsivity and distractibility), associated with deficient goal-directed behavior. Frontal lobe lesions in adult humans often leads to hyperactivity/hyperreactivity. In childhood, however, we are likely looking at ADHD as a problem of the trajectory of normal maturation of the frontal lobes with effects on the direct, indirect and/or hyperdirect pathways.

## Data availability statement

The original contributions presented in this study are included in the article/supplementary material, further inquiries can be directed to the corresponding author.

## Author contributions

Both authors shared equally in preparation of the manuscript, contributed to the article, and approved the submitted version.
